# Effects of the Backbone’s Structures on the Curing Behaviors and Properties of Phthalonitrile Containing Benzoxazine Rings

**DOI:** 10.3390/molecules29235637

**Published:** 2024-11-28

**Authors:** Mingzhen Xu, Jiaqu Zhang, Bo Li, Zexu Fan, Lunshuai He

**Affiliations:** 1 Yangtze Delta Region Institute (Huzhou), University of Electronic Science and Technology of China, Huzhou 313001, China; 2 School of Materials and Energy, University of Electronic Science and Technology of China, Chengdu 610054, China

**Keywords:** phthalonitrile, curing behaviors, thermal stability, mechanical properties

## Abstract

Phthalonitrile-based resins and benzoxazine play important roles in the field of advanced materials because of their excellent properties. In order to understand the effect of the backbone’s structure on the curing kinetics and properties of the multifunctional resin matrices, different kinds of phthalonitrile containing benzoxazine with various backbone structures were designed and prepared. The curing processes and curing behaviors were investigated by differential scanning calorimetry (DSC). With the assistance of the orthogonal test analysis method, the kinetic parameters, including activation energy *E_α_*, were evaluated and calculated. Results indicated that an autocatalytic model for the curing reaction of various phthalonitrile−containing benzoxazine resins was confirmed. Nevertheless, the activation energies for reactions of benzoxazine and nitrile groups were significantly changed due to the steric hindrance derived from the backbone’s structures. The thermal stability of polymers cured at various temperatures was evaluated by TGA testing. Then, their mechanical properties were investigated and confirmed with SEM images of fracture surfaces. Also, the thermal expansion characteristics of the various polymers were investigated. Results demonstrated that this work proposed an improved matrix resin system with outstanding thermal stability and mechanical properties that broadened the foundation and ideas for subsequent research.

## 1. Introduction

The phthalonitrile-based resin containing benzoxazine (BA−ph) is a kind of high-performance thermosetting resin. Excellent properties such as high-temperature resistance, high glass transition temperature, excellent thermal stability and mechanical properties, low water absorption, high flame retardancy and almost zero shrinkage reinforce the position of this resin as a favored choice in the high-performance composite materials industry. Compared with the conventional nitrile resin, which requires high curing temperature, long curing time and extra catalyst, BA−ph shows the characteristics of self-catalytic polymerization due to the existence of an oxazine ring [1,2,3]. Chen and co-authors reported a benzoxazine containing a fluorinated aromatic ether nitrile linkage, which exhibits outstanding structural strength and dielectric properties and is appropriate for use in shipbuilding and electronic packaging materials [4]. Li and co-authors carried out research to study the effect of catalysts on the polymerization mechanism and thermal properties of benzoxazine-containing nitrile resin. The findings indicated that the introduction of catalysts notably reduced the formation temperature of the polymer, which made the polymer anticipated to be applicable in the domain of flame retardant and heat-resistant materials [5]. Wu and co-authors prepared a kind of nitrile-functionalized benzoxazine/bismaleimide blend and its glass cloth-reinforced laminates. The application of the laminated composite in temperature-resistant structural materials was explored [6]. In our previous work, it was certificated that the monomer of BA−ph contains active functional groups, amino and phenolic groups, and has a unique two-stage polymerization mechanism. That is, under the heating condition, the oxazine ring undergoes ring-opening polymerization to form the active phenolic Mannich bridge structure, which can effectively promote the ring-forming polymerization of nitrile groups and obtain a polymer system with excellent thermal stability and outstanding structural strength [7]. However, the main chain structure, steric hindrance, substituents and other factors can significantly affect the ring-opening efficiency and degree of benzoxazine ring. Moreover, the ring-opening form of the oxazine ring will directly affect the ring-forming reaction rate and polymer aggregation state and then affect the macro properties of the polymer [8,9]. Therefore, the study of the effect of its structure and polymerization behavior on the properties of cyanopolymer and the mechanism of the influence of structure and crosslinking reaction process on the properties of the polymer can lay a theoretical and experimental foundation for the development and the optimization of the existing polymer system.

For the thermosetting resins, the influence of structure on the properties is mainly achieved by influencing the polymerization process and behaviors. The curing reaction is a very complex process because many reactive processes occur simultaneously [10,11]. Also, the final properties of the crosslinked polymers significantly depend on the curing kinetics of the polymerizations concerned with the extent of curing degree, the curing conditions and so on [12,13]. Therefore, the study of curing kinetics contributed to both a better knowledge of process development and the nature of its curing process, the structure of the cured material, and how its kinetic parameters can be influenced by temperature, etc. Several techniques have been utilized to examine the curing kinetics of phthalonitrile-based resins, including differential scanning calorimetry (DSC) [14,15,16,17], Fourier transform infrared spectroscopy (FTIR) [10] and rheokinetic measurements [18,19]. Among these, the DSC techniques have been the most utilized methods to determine the curing kinetics parameters and rate equations of the polymerization of phthalonitrile-based resins [17,20,21]. In general, the kinetics parameters estimated from DSC dynamic experiments were reported to agree well with those estimated by other techniques. As a precise measure, the non-isothermal DSC method has been carried out at various heating rates to evaluate the curing kinetic parameters [10,22]. Due to the fact that the kinetic data can be obtained in a relatively short period of time, the method has attracted increasing interest [11,23]. Therefore, in this work, the curing kinetics of phthalonitrile-based resin containing benzoxazine was studied via the non-isothermal DSC technique, assisted with the peak-fitting method.

Herein, in order to deeply understand the influence of molecular structure on polymerization behavior and process, a new benzoxazine containing nitrile-resin (WZ−cn) with fluorene structure was synthesized through molecular structure design and verified by nuclear magnetic and infrared structural characterization methods. At the same time, the curing reaction processes of BA−ph and WZ−cn were compared and characterized by DSC, the reaction types were determined by equal conversion analysis, and the activation energy of the system reaction was obtained. The influence of molecular structure on reaction behavior, process and kinetic parameters was compared. Finally, the curing and molding temperature range of benzoxazine nitrile-based resin containing fluorene was calculated by the extrapolation method. It provides basic data for the subsequent preparation of high-performance polymer systems. The thermal stability of the BA−ph and WZ−cn polymers was also investigated. Also, the mechanical properties were discussed and supported by the SEM.

## 2. Results and Discussion

### 2.1. Structural Characterization

**Figure 1:**
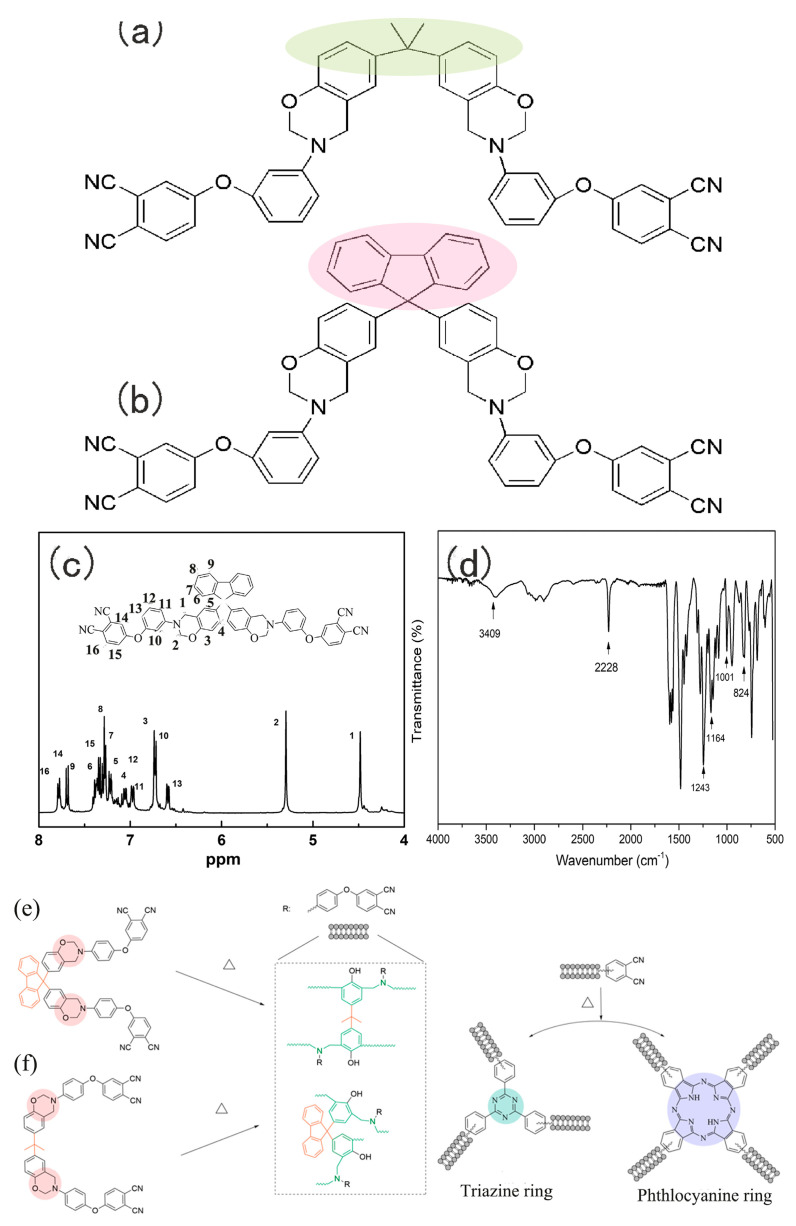
Structure of (**a**) BA−ph and (**b**) WZ−cn, (**c**) ^1^HNMR spectrum of WZ−cn, (**d**) FTIR spectrum of WZ−cn, (**e**,**f**) the possible polymerization of WZ−cn and BA−ph.

Figure 1 presents the structures of BA−ph and WZ−cn. To verify the target structure of WZ−cn, the proton nuclear magnetic resonance spectrum (^1^H-NMR) and Fourier transform infrared (FTIR) spectrum are shown in Figure 1c and Figure 1d, respectively. Compared with the structure of BA−ph verified in our previous literature [24], Figure 1c highlights the characteristic absorption bands of WZ−cn. By referring to the standard spectra and analyzing the types of hydrogen in the molecular structure of WZ−cn, it can be known that the characteristic peaks respectively correspond to the characteristic protons on the oxazine ring and protons at different positions in different benzene rings. Among them, the aromatic protons are concentrated within the range of 6.69–7.79 ppm [25]. The two distinct hydrogens on the benzoxazine ring are concentrated at 4.48 ppm (N-CH_2_-Ar) and 5.29 ppm (N-CH_2_-O), respectively, indicating the successful synthesis of the main structure of the fluorene-containing benzoxazine cyanide resin monomer [4,6]. Through normalization fitting and comparing the differences in the intensities of the characteristic peaks of protons at different positions, taking the two protons on the oxazine ring as an example, it can be found that there is a slight difference in the integrated areas of the two protons, suggesting that there exists a small amount of pre-polymer structure with ring opening in the synthesis product. The form of ring opening of the pre-polymer mainly exists in the form of phenolic hydroxyl groups.

Figure 1d presents the infrared spectrum of the WZ−cn resin. The absorption peak at 2230 cm^−1^ is the characteristic stretching vibration peak of −CN [4,24]. The characteristic absorption peaks of the C−N−C structure in the benzoxazine ring occur at 1169 cm^−1^ and 824 cm^−1^ (stretching vibration), and the characteristic absorption peaks of the C−O−C structure occur at 1243 cm^−1^ and 1002 cm^−1^ (stretching vibration) [24]. This indicates that the main functional groups and structure of the fluorene-containing benzoxazine cyanide resin conform to the design [7,8,24]. Through the aforementioned synthesis method, the target product with relatively high purity can be obtained.

### 2.2. Curing Behaviors and Reaction Kinetics of BA−ph and WZ−cn

**Figure 2:**
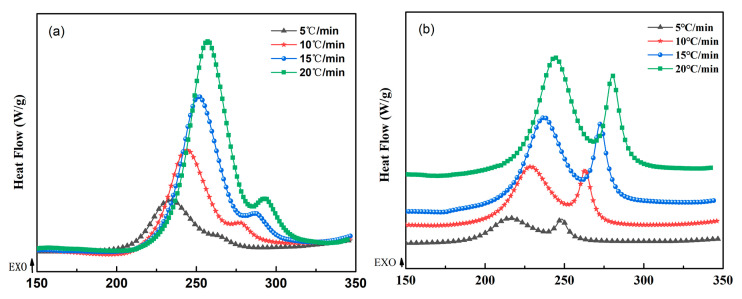
DSC curves of (**a**) WZ−cn and (**b**) BA−ph.

To obtain the basic experimental data for the analysis of polymerization reaction kinetics, the reaction processes of the WZ−cn and the BA−ph monomer were tested and characterized through DSC, shown in Figure 2. The polymerization reaction behavior of the resin system was monitored under different heating rates to acquire the influence of the temperature effect on the polymerization reaction process [7,23]. Then, basic formulas were utilized for analysis and fitting to obtain in-depth polymerization reaction kinetics rules. Figure 2a,b, respectively, present the DSC curves of the WZ−cn monomer and BA−ph monomer at heating rates of (5 °C/min, 10 °C/min, 15 °C/min, and 20 °C/min). It can be observed from the figures that distinct double curing exothermic peaks are respectively presented in the DSC curves of WZ−cn and BA−ph, indicating that the curing reaction of the resin system belongs to a double curing reaction, which respectively corresponds to the ring-opening polymerization of the benzoxazine ring and the ring-closing polymerization of the nitrile group [26]. It can be clearly seen from Figure 2a that as the heating rate increases, the enthalpy of the curing exothermic peak significantly increases, and the peak temperature of curing shifts to a higher temperature. This is because as the heating rate increases, the environmental temperature changes more rapidly. At this time, heat conduction lags, making it more difficult for the thermal motion of molecules to follow the increase in the environmental temperature. Moreover, the molecules enter the high-temperature range more rapidly, causing incomplete conversion at lower temperatures and a decrease in the conversion rate, thereby shifting the exothermic peak to the right [26]. In the higher temperature range, because the resin system is sensitive to temperature, the curing reaction rate significantly increases, and the corresponding curing exothermic enthalpy significantly increases, making the reaction peak sharper.

By comparing Figure 2a,b, it can be clearly seen that there are significant differences in the peak shapes of the curing reaction exothermic peaks of the WZ−cn and BA−ph. In Figure 2b, the heights of the double curing exothermic peaks show similar enthalpy, while the height of the second curing exothermic peak in Figure 2a significantly decreases. According to the above analysis, the double curing exothermic peaks respectively correspond to the ring-opening reaction of the benzoxazine ring and the ring-forming reaction of the nitrile group [19,23,24]. Therefore, in the DSC test results of the WZ−cn, the decrease in the exothermic peak of the ring-forming polymerization of the nitrile group indicates that under the same curing conditions, the polymerization reaction rate and ring-forming polymerization efficiency of the nitrile group are significantly lower than those of the bisphenol A-type resin system [27,28]. Previous studies have shown that in the absence of a catalyst, the ring-forming polymerization of the nitrile group in the benzoxazine nitrile resin system is mainly initiated and promoted by the active phenolic hydroxyl groups generated from the ring-opening polymerization of the benzoxazine ring and the lone pair electrons in the structurally regular imine structure [23]. Combined with the phenomenon that the enthalpy of the exothermic peak corresponding to the ring-opening reaction of the benzoxazine ring is relatively large in the DSC test of the WZ−cn, it can be indicated that the ring-opening reaction of the benzoxazine ring is basically not affected by the fluorene structure of the main chain. Therefore, the decrease in the enthalpy of the ring-forming polymerization exothermic peak of the nitrile group can be attributed to the fact that the phenolic hydroxyl groups and active imine structures generated by the ring-opening of the fluorene-containing benzoxazine cannot efficiently stimulate the ring-forming reaction of the nitrile group due to the steric hindrance of the main chain, thereby resulting in a lower polymerization reaction efficiency of the nitrile group.

Table 1 respectively shows the peak temperature ranges of the curing reactions of the WZ−cn monomer and the BA−ph monomer. The peak temperature range of the first curing peak of WZ−cn is 232 °C to 259 °C, and the peak temperature range of the second curing peak is 265 °C to 295 °C. While the peak temperature range of the first curing peak of BA−ph is 216 °C to 244 °C, and the peak temperature range of the second curing peak is 248 °C to 281 °C. This indicates that at the same heating rate, the curing reaction rate of WZ−cn is slower than that of the BA−ph resin monomer; that is, the curing difficulty of WZ−cn is greater, and a higher temperature is required to initiate the reaction. This difference may be due to the presence of the fluorene structure in WZ−cn, which leads to an increase in the steric hindrance of the system [24]. The fluorene structure makes it more difficult for the chain segments that need to undergo polymerization during the curing process to achieve the ideal arrangement, thereby requiring an increase in the reaction temperature to overcome the steric hindrance effect and promote the reaction [29].

**Figure 3:**
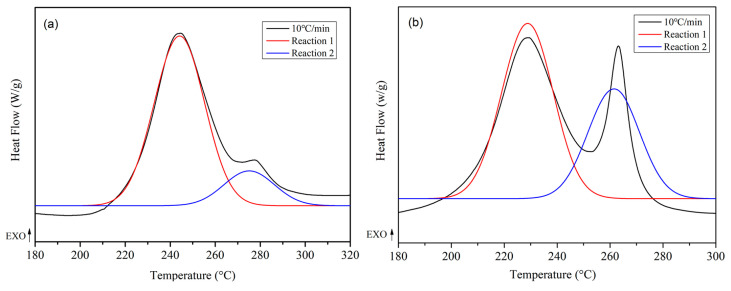
Peak separation fitting of the DSC curve with a heating rate of 10 °C/min, (**a**) WZ−cn and (**b**) BA−ph.

To further investigate the reaction kinetics of the system, the two-step reaction process was decomposed into two individual one-step reactions through peak separation fitting, and the reaction process was analyzed. The DSC curves of the curing reactions of the WZ−cn and the BA−ph monomer were processed by peak separation fitting using PeakFit v4.12 software, with a fitting goodness fit of r^2^ = 0.95. The obtained results are presented in Figure 3a,b, taking the DSC curves of the curing reactions of the WZ−cn monomer and the BA−ph monomer at a heating rate of 10 °C/min as examples. The results of the peak separation of the curing reactions were respectively designated as Reaction 1 and Reaction 2.

In conclusion, when comparing factors such as the initial reaction temperature, peak curing temperature, reaction enthalpy and the shape of the polymerization exothermic peak of the fluorene-containing benzoxazine nitrile resin and the bisphenol A-type benzoxazine nitrile resin, it can be observed that the introduction of the fluorene structure in the main chain does not alter the fundamental polymerization mode of the double curing reaction of the benzoxazine nitrile resin monomer [17,23]. Nevertheless, the incorporation of the fluorene structure slightly increases the difficulty of ring-opening of the benzoxazine ring, resulting in a slightly elevated temperature for the ring-opening polymerization reaction. Additionally, the introduction of the fluorene structure leads to a sharp increase in the spatial steric hindrance of the main chain structure, preventing the active phenolic hydroxyl groups and imine structures generated by the ring-opening of the oxazine ring from stimulating the ring-closing polymerization reaction of the nitrile group in the optimal arrangement, thereby significantly inhibiting the polymerization reaction of the nitrile group [27].

### 2.3. Curing Behaviors and Reaction Kinetics of BA−ph and WZ−cn Monomers

**Figure 4:**
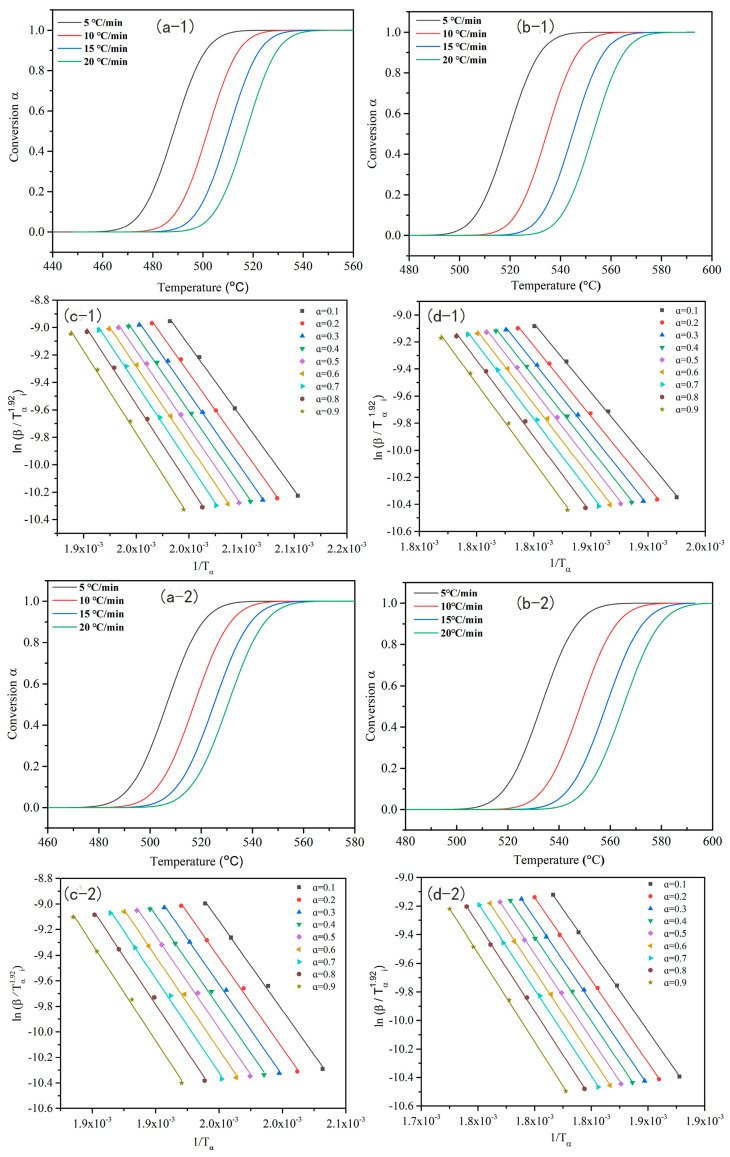
Curves of conversion rates α as function of curing temperatures for BA−ph: (**a**−**1**) Reaction 1, (**b**−**1**) Reaction 2; straink plots at various conversion of BA−ph reaction: (**c**−**1**) Reaction 1, (**d**−**1**) Reaction 2; curves of conversion rates α as function of curing temperatures for WZ−cn: (**a**−**2**) Reaction 1, (**b**−**2**) Reaction 2; straink plots at various conversion of WZ−cn reaction: (**c**−**2**) Reaction 1, (**d**−**2**) Reaction 2.

The reactions corresponding to the two curing peaks in Figure 2 and Figure 3 were named Reaction 1 and Reaction 2, respectively. The DSC data at these four different heating rates (shown in Figure 2) were used to calculate the change in monomer conversion with temperature, as shown in Figure 4. In Figure 4(a−1,b−1,a−2,b−2), all curves possessed a typical sigmoid shape, which indicated that the curing reaction of BA−ph and WZ−cn followed the autocatalytic mechanism. So, the isoconversional method was adopted, and Starink Formula (1) [30] was used to evaluate the activation energy ($E_{\alpha }$) and polymerization type of BA−ph and WZ−cn, respectively:

(1) $$ln(\frac{\beta_{i}}{T_{\alpha ,i}^{1.92}})=Const-1.0008(\frac{E_{\alpha }}{RT_{\alpha }})$$

$ln(\beta_{i}/T_{\alpha ,i}^{1.92})$ was plotted against $1/T_{\alpha }$, the dependence of conversion rate (α) on temperature (T) shows a good linear relationship, as shown in Figure 4(c−1,d−1,c−2,d−2). The slope of the straight line in Figure 4c,d is $-1.0008E_{\alpha }/R$, from which the relationship between the activation energy ($E_{\alpha }$) and α can be obtained.

Assuming that under the test condition of a heating rate of 10 °C/min, the monomer can be fully cured, the total enthalpy in the curve represents a polymerization reaction conversion rate of 100%. Based on this premise, the polymerization reaction conversion rates of the WZ−cn monomer and the BA−ph monomer at different polymerization temperatures can be calculated through fitting. Further, the relationship between the conversion rate of the polymerization reaction and temperature can be obtained through curve fitting, and the resulting curves are shown in Figure 4. By observing the forms of the fitted curves for the ring-opening polymerization reaction of benzoxazine and the ring-closing polymerization reaction of the nitrile groups, it can be seen that the polymerization reaction process curves of the WZ−cn monomer and the BA−ph monomer after peak separation processing all exhibit an “S” shape, indicating that their polymerization reaction processes belong to a single self-catalytic reaction process [31,32]. Simultaneously, this also indicates that the peak separation fitting process is relatively reasonable. Additionally, by comparing the curve forms of the benzoxazine nitrile resin containing a fluorene structure and the bisphenol A-type benzoxazine nitrile resin, it can be discovered that the introduction of the fluorene structure does not alter the self−catalytic reaction mechanism followed by the curing reaction.

It is known from the above that the slope of the fitted straight line is $-1.0008E_{\alpha }/R$. Given that R is a constant, it is found in Figure 4(c−1,d−1,c−2,d−2) that the slope of the straight line does not fluctuate significantly, which indicates that both Reaction 1 and Reaction 2 can be represented by a one-step reaction model. Under normal conditions, the activation energy of the polymerization reaction is approximately constant. Therefore, the value of the activation energy can be obtained by averaging the values of the activation energy at different conversion rates. The results of the activation energy of the WZ−cn and BA−ph monomer polymerization reactions obtained are shown in Table 2.

According to Table 2, it can be observed that by comparing the activation energies of the polymerization reactions of the WZ−cn monomer and the BA−ph monomer, the $E_{\alpha }$ of the ring-opening polymerization reaction of the benzoxazine ring corresponding to Reaction 1 is significantly greater than that of the ring-forming polymerization reaction of the nitrile group corresponding to Reaction 2. This implies that the ring-opening reaction process of the oxazine ring is more susceptible to temperature variations. Therefore, the curing reaction can be initiated more efficiently by increasing the curing reaction temperature [33]. Additionally, the polymerization reaction of the nitrile functional groups in traditional o-phthalonitrile resins is rather difficult to occur spontaneously and typically requires the introduction of a catalyst. It has been reported in the literature [34] that under the condition of introducing ZnCl_2_, the activation energy of the nitrile group reaction in o-phthalonitrile is 108 kJ·mol^−1^. In this research work, regardless of whether it is the bisphenol A-type benzoxazine nitrile resin (BA−ph) or the fluorene-containing benzoxazine nitrile resin (WZ−cn), the apparent activation energies of their nitrile group polymerization reactions are both less than 108 kJ·mol^−1^, suggesting that the ring-opening of benzoxazine has a better-promoting effect on the ring formation of the nitrile group. Furthermore, the activation energies of both the ring-opening reaction of the benzoxazine ring and the ring-closing polymerization reaction of the nitrile group of the WZ−cn monomer are higher than those of the BA−ph monomer. This indicates that the presence of the fluorene structure in the main chain results in a significant steric hindrance among the active functional groups, thereby increasing the difficulty of the thermal polymerization reaction. Among them, the activation energy of the ring-forming polymerization reaction of the nitrile group of the WZ−cn is significantly higher than that of the BA−ph, which also demonstrates that the presence of the fluorene structure limits the initiation and promotion of the ring-closing polymerization of the nitrile group by the ring-opened benzoxazine structure. This conclusion is consistent with the phenomenon in the previous DSC test results, where the heat enthalpy of the second curing reaction exothermic peak of the WZ−cn is significantly reduced.

### 2.4. Curing Processes of BA−ph and WZ−cn Monomers

**Figure 5:**
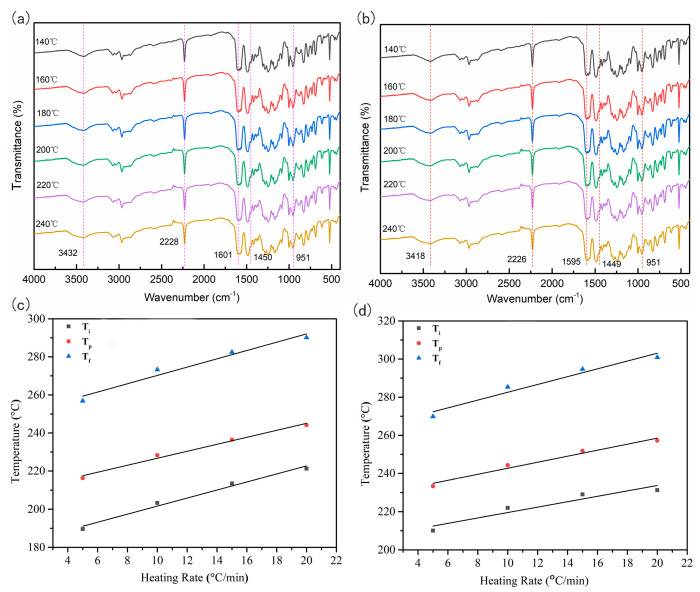
In situ FTIR spectra and non-isothermal curing temperature rise rate relationship curves of WZ−cn and BA−ph, (**a**,**c**) WZ−cn, (**b**,**d**) BA−ph.

Through in situ infrared spectroscopy, the changes in functional groups and related structures of the WZ−cn and the BA−ph monomer during the alteration of curing temperature were characterized. After normalization of the obtained curves, the results are presented in Figure 5. It can be distinctly observed from Figure 5a,b that when the temperature rises to 160 °C, the intensity of the benzoxazine absorption peak at 951 cm^−1^ begins to weaken. The absorption peak near 1601 cm^−1^ is for the 1, 2, 4−trisubstituted benzene ring [29]. As the temperature increases, the peak area begins to decrease, and the characteristic peak of the Mannich bridge at 1450 cm^−1^ increases, all of which indicate the occurrence of the ring−opening polymerization of the benzoxazine ring. When the temperature rises to 200 °C, the characteristic absorption peak of the nitrile group near 2228 cm^−1^ also starts to weaken, which is associated with the consumption of the nitrile functional group. However, until the end of the treatment at 240 °C, there is still a prominent sharp peak at this characteristic absorption peak, indicating that the nitrile functional group has strong inertness and is difficult to cure. The stretching vibration peak of the phenolic hydroxyl group near 3432 cm^−1^ gradually broadens as the treatment temperature rises, suggesting that the phenolic hydroxyl group participates in the polymerization reaction of the system during curing and forms intramolecular or intermolecular hydrogen bonds [20]. By comparing Figure 5a,b, it can also be found that the introduction of the fluorene structure does not change the ring−opening reaction of the benzoxazine ring and the ring-forming polymerization process of the nitrile functional group. Furthermore, under the same test conditions, the characteristic functional groups of the two types of benzoxazine cyano resins exhibit similar changes, indicating that within this temperature range, the fluorene structure in the main chain and the bisphenol structure are insufficient to significantly alter the progress of the polymerization reaction.

To obtain high-performance polymers with outstanding comprehensive properties, the curing and molding of thermosetting resins are typically conducted using a stepped temperature increase approach [30]. During the curing process, the temperature selection at different stages is associated with the initial temperature of the curing reaction (*T_i_*), the peak temperature of the first curing peak (*T_p_*) and the terminal temperature (*T_f_*). The polymerization reaction process of resin monomers and curing reaction peaks can be initially observed through DSC curves. However, the curing peaks are influenced by factors such as the heating rate, which leads to difficulty in determining the actual curing temperature of thermosetting resins. In this work, based on the research of polymerization reaction kinetics and the thermal analysis data obtained from DSC tests, the curing and molding process conditions of the WZ−cn will be further determined, providing a theoretical basis for the preparation of high-performance benzoxazine cyano resin polymers.

There exists a linear relationship between the curing reaction temperature (*T*) and the heating rate (*β*) during the curing process [18]. Thus, through DSC curves at multiple different heating rates, using the linear relationship between *T* and *β*, the curing and molding temperature can be determined by extrapolation. In engineering, the extrapolation method using the *T*−*β* diagram is employed to obtain the curing process temperature parameters, and then the optimal process temperature is determined through experiments. The extrapolation analysis was carried out on the DSC test results of the WZ−cn and the BA−ph at different heating rates, and the process temperatures and extrapolated characteristic temperatures of the corresponding resin monomers during the curing process are listed in Table 3 and Table 4. It can be observed from the data in the tables that the extrapolated curing initial temperatures of the WZ−cn monomer and the BA−ph monomer are within the range of 180–220 °C, and the post-curing temperatures are within 240–270 °C. If the curing is divided into two stages, the temperature between the extrapolated T_i_ and the extrapolated *T_p_* is generally selected as the curing process temperature, while the temperature between the extrapolated *T_i_* and the extrapolated *T_f_* is used as the post-curing temperature. If the curing is divided into three stages, the three extrapolated characteristic temperatures can be directly used as the process temperatures for the curing process. The extrapolation method can obtain the approximate curing process temperature of the system.

### 2.5. Thermal Stability and Mechanical Properties of BA−ph and WZ−cn Polymers

**Figure 6:**
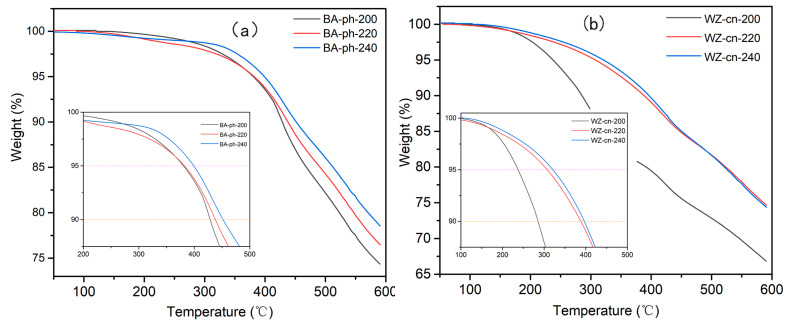
TGA curves of (**a**) BA−ph and (**b**) WZ−cn.

Figure 6a,b illustrate the thermal stability of BA−ph and WZ−cn polymers obtained at various curing temperatures. The TGA analysis curves are presented in Figure 6, and the corresponding thermal performance data are listed in Table 5, including *T*_5%_ (the decomposition temperature at 5% mass loss), *T*_10%_ (the decomposition temperature at 10% mass loss) and *Yc* (the char yield at 600 °C).

As per Figure 6a and Table 5, the *T*_5%_ of the BA−ph polymer treated at different curing temperatures is all above 380 °C. The *T*_10%_ ranges from 428 °C to 452 °C, and the *Yc* at 600 °C is greater than 74%, suggesting that the cured samples possess excellent thermal stability. According to Table 5 and Figure 6b, the curing degree of WZ−cn−200 is extremely low. The *T*_5%_ is merely 237.39 °C, and the *T*_10%_ is lower than 300 °C, indicating that the curing degree of WZ−cn at this temperature is relatively low and it is insufficient to be classified as a heat-resistant polymer. Simultaneously, as the curing temperature rises, the *T*_5%_, *T*_10%_ and *Yc* of the cured samples all increase. When the post-curing temperature reaches 240 °C, the thermal performance data of BA−ph−240 is the most favorable, with *T*_5%_ approaching 400 °C, *T*_10%_ at 451.71 °C and *Yc* at 78.59% at 600 °C. The reason for the superior thermal stability of BA−ph−240 is that the higher curing temperature promotes the self-polymerization reaction of BA−ph, enhancing the crosslinking degree of the system. When comparing the heat resistance of WZ−cn and the BA−ph under the same curing conditions, the thermal stability of WZ−cn is significantly lower than that of BA−ph. This is because the presence of the rigid group with a fluorene structure leads to a significant steric hindrance effect, which greatly inhibits the polymerization reaction of the active functional groups and reduces the degree of polymerization of the system [24,34]. Although the introduction of the rigid group can, to a certain extent, enhance the high−temperature resistance of the polymer system, its inhibitory effect on the polymerization reaction is more pronounced, resulting in a macroscopic decrease in the heat resistance of the polymer system. The mass loss of benzoxazine nitrile resin containing bisphenol fluorene may be related to the following factors: (1) The Impact of Curing Behavior: Based on the research, the curing behavior of benzoxazine resin is affected by the position of substituents. Bisphenol fluorene, as a specific substituent, influenced the curing reaction of benzoxazine resin, thereby influencing its thermal stability and quality retention rate. Benzoxazine resins containing bisphenol fluorene might display lower thermal stability during heat treatment due to their structural characteristics, leading to considerable mass loss [27]; (2) Changes in Crosslinking Density: The introduction of bisphenol fluorene may modify the crosslinking density of the resin. Due to the inhibition of polymerization, the introduction of bisphenol fluorene reduces the crosslinking density, it may cause a decline in thermal stability and, consequently, greater mass loss during heat treatment [35]; (3) Differences in Pyrolysis Mechanisms: Benzoxazine resins with different structures may demonstrate distinct mechanisms during the pyrolysis process. Benzoxazine resins containing bisphenol fluorene may be more prone to molecular chain cleavage during pyrolysis due to the ring-opening of fluorene rings, resulting in significant mass loss [36].

**Figure 7:**
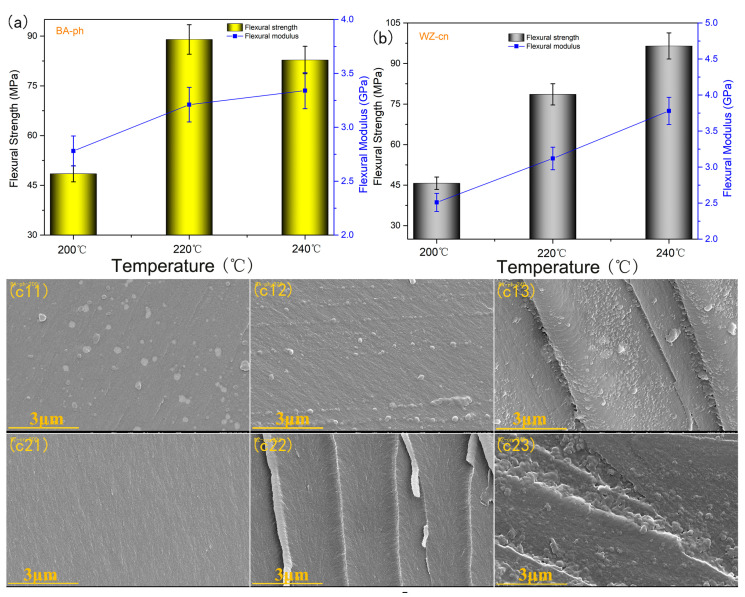
Flexural strength and modulus of (**a**) BA−ph, (**b**) WZ−cn and SEM images of BA−ph and WZ−cn, (**c11**) BA−ph−200, (**c12**) BA−ph−220, (**c13**) BA−ph−240, (**c21**) WZ−cn−200, (**c22**) WZ−cn−220, (**c23**) WZ−cn−240.

Figure 7 delineates the flexural strength and flexural modulus of BA−ph and WZ−cn polymers, revealing a progressive increase and then decline in flexural strength as the curing temperature rises. This attenuation in toughness is likely attributed to the increased crosslinking density. Conversely, the flexural modulus of the polymer resin exhibits a durable increase. This behavior can be ascribed to intermolecular crosslinking polymerization, which endows the resin with enhanced intermolecular forces through its polar nitrile groups and uniformly augments the crosslinking density. Consequently, the polymer retains substantial strength while achieving a notably elevated modulus. Nevertheless, during the network formation, an imperfect crosslinking structure induces a significant brittleness, leading to a pronounced reduction in flexural strength, as shown in Figure 7a. As the curing temperature escalates, the flexural modulus of all the polymer resin exhibits a trend of increase. This phenomenon occurs as the nitrile groups progressively engage in polymerization reactions with rising temperatures.

The above discussions focused on the polymerization reaction process and its influence on the flexural strength and modulus of different structural benzoxazine nitrile−based resins under heat treatment conditions. In order to further study the influence of the polymerization reaction process on the molecular aggregation morphology and the relationship between the aggregation morphology and the final polymer thermal performance, scanning electron microscopy (SEM) was used to observe the changes in the microscopic morphology of the two different structural benzoxazine nitrile-based resin polymers under different curing conditions [37,38]. The morphological characterization results are shown in Figure 7c. From the figures, it can be seen that the cross-sectional morphology of the polymer is significantly different under different curing conditions. For the same polymer, as the curing temperature increases, the cross-sectional morphology of the polymer becomes rougher, and the surface begins to have granular structures. Combining the analysis above, this may be because the resin system underwent segmented polymerization, with the benzoxazine ring self-polymerizing to form tight local agglomeration structures, while the nitrile-based group underwent ring polymerization to form relatively dense aromatic heterocyclic structures. Due to the segmented polymerization, the microscopic structure appears to have an uneven and discontinuous granular distribution. Comparing the two structural benzoxazine nitrile-based resin polymers, BA−ph−200 has already begun to form granules on its surface at lower curing temperatures, while WZ−cn−200’s surface remains relatively smooth, which also agrees with the DSC test results, where WZ−cn’s curing starting temperature is higher than that of BA−ph. At the same time, as the curing temperature increases, the cross-sectional fracture surface of the polymer begins to show a feathered fold, which is due to the stress transmission from being affected by the internal molecular aggregation morphology of the polymer during the curing of the highly crosslinked network structure resin, resulting in the transmission path appearing to twist multiple times [39,40,41]. Compared with BA−ph, the dendritic section of WZ−cn begins to appear after curing at 220 °C. This is due to the introduction of a fluorene structure, which improves the rigidity of the molecular chain. The increase in rigidity will lead to irregular aggregation of molecular chain segments, and the obstruction effect on stress transmission will be enhanced accordingly, which is manifested as dendritic fracture in the section morphology.

**Figure 8:**
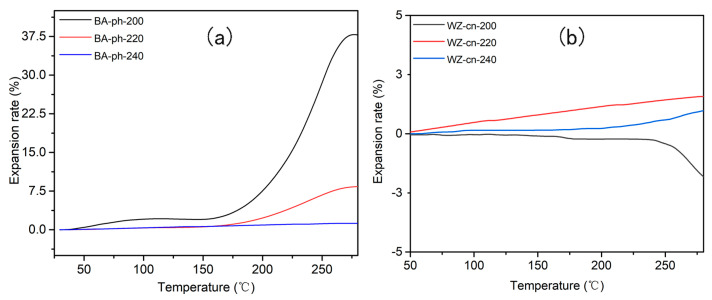
TMA curves of (**a**) BA−ph and (**b**) WZ−cn.

To assess the dimensional stability of polymers under diverse heat treatment temperature conditions, we employed static thermomechanical property testing (TMA) to obtain the thermal expansion rate and the coefficient of linear thermal expansion (CTE) of the polymers. In this section, the thermal expansion rate at 250 °C and the CTE values within the range of 50 °C to 250 °C were selected to evaluate the static thermomechanical properties of WZ−cn and BA−ph. The results of the TMA test are presented in Figure 8, and the detailed data are recorded in Table 6. The calculation of CTE is derived from Formula (3):

(2) $$\mathrm{CTE}=\frac{\Delta L}{L\times \Delta T}$$

In the equation, ∆*L* denotes the thickness variation of the resin within the selected temperature range; *L* represents the initial thickness of the resin, and ∆*T* represents the value of the selected temperature range.

The thermal expansion behavior of materials is intimately associated with its application. For instance, materials featuring low or negative thermal expansion can be employed to fabricate components that are dimensionally stable under temperature variations, which is crucial for the stability of precision instruments and structures [42]. Negative thermal expansion materials can also be utilized to manufacture composites, and by combining them with positive thermal expansion materials, near-zero net thermal expansion can be attained, which is highly valuable for certain specific engineering applications. The expansion rate of a material, be it positive or negative, is associated with its thermal expansion behavior. A positive expansion rate indicates that the material will increase in volume or length upon heating, whereas a negative thermal expansion rate (NTE) implies that the material will contract upon heating. This phenomenon is of great significance in materials science as it affects the properties and applications of materials at varying temperatures. In this work, the negative expansion coefficient of the WZ−cn−200 sample is primarily ascribed to the secondary curing contraction of the sample under the test temperature circumstances. Grounded on the aforementioned analysis, the presence of fluorene structure within WZ−cn can inhibit the polymerization of resin monomer. After formation at 200 °C, the degree of curing of the composite system is relatively low, and there remains a substantial number of unreacted active functional groups in the system. These functional groups will persist in crosslinking under the secondary heating condition, inducing the resin to manifest a shrinkage phenomenon and, subsequently, a negative expansion coefficient. The thermal expansion coefficient of BA−ph−200 is positive because the reactivity of BA−ph is conspicuously higher than that of WZ−cn. After heat treatment at 200 °C, the system has demonstrated a higher degree of crosslinking, and the polymer system will not undergo shrinkage due to the crosslinking reaction under the test conditions.

It can be intuitively perceived from Table 6 that the thermal expansion rate of the WZ−CN system is extremely lower than that of BA−ph. This is primarily because of the following reasons. (1) The introduction of the fluorene structure significantly enhances the rigidity of the polymer chain. The bulky nature of the fluorene structure restricts the internal rotation and thermal motion of the polymer chain segments, thereby raising the glass transition temperature and inherent thermal stability. (2) When polymerizable groups are present in the fluorene-based benzoxazine structure, it enhances the crosslinking density of the polymer. This augmented crosslinking density contributes to the improvement of the initial thermal decomposition temperature and char yield of the polymer. Simultaneously, the glass transition temperature is also significantly elevated, which may lead to a stable thermal expansion rate. (3) During the curing process of benzoxazine resins, the introduction of fluorene potentially forms a more uniform crosslinked network structure. This uniformity helps sustain consistent thermal expansion behavior at different temperatures, thereby enabling a constant thermal expansion rate.

In conclusion, the benzoxazine-based resin containing the fluorene structure demonstrates a constant thermal expansion rate from the beginning of the test primarily because the introduction of the fluorene structure enhances the rigidity of the polymer, increases the crosslinking density, and improves the thermal stability and heat resistance of the material. These factors collectively contribute to the stability of the thermal expansion behavior of the resin.

## 3. Materials and Methods

### 3.1. Materials

The phthalonitrile-based resin containing benzoxazine based on bisphenol A (BA−ph) and bisphenol fluorene (WZ−cn), formaldehyde and 3-aminophenoxyl-o-phthalonitrile was synthesized with 1,4-dioxide and methylbenzene as the solvent. The synthesis process is referenced with minor adjustments [18,43]. Bisphenol A, bisphenol fluorene and formaldehyde were obtained from Chengdu Kelong Chemicals Co. Ltd., Chengdu, China. 1,4-dioxide and methylbenzene were purchased from Shanghai Bodi Chemical Co. Ltd., Shanghai, China. Aminophenoxy phthalonitrile (APN, Tm = 174 °C) was prepared and purified in our lab [17]. All reagents were of analytical grade and used without further purification. The chemical structures of BA−ph and WZ−cn are shown in Figure 1.

### 3.2. Preparation of BA−ph and WZ−cn Polymers

In a glass flask with three necks equipped with a mechanical stirrer, BA−ph and WZ−cn resins were introduced and immersed in an oil bath, respectively. Stirring was initiated at a rate of 290 to 310 revolutions per minute (rpm), and the oil bath temperature was set to 140 to 150 °C, with heating commenced. The pre-polymerization was allowed to proceed for one hour. Subsequently, the stirring speed was reduced to 90 to 110 rpm and maintained for an additional half-hour. Then, the resultant pre-polymer was poured into a foil-lined tray and subjected to a vacuum oven, set at 170 °C, where a vacuum of −0.08 to −0.10 megapascals (MPa) was established and sustained for a duration of 30 min. Subsequently, a programmed heating sequence was initiated within the vacuum oven, progressing through a series of temperatures and durations: 180 °C−1 h/200 °C−2 h/220 °C−2 h/240 °C−2 h. Upon completion of the thermal program, the copolymerization resin was allowed to cool gradually within the vacuum oven, removed only once the temperature had descended below 50 °C. The resultant copolymerized resins were subsequently sectioned into appropriate dimensions for mechanical property evaluation. The nomenclature for the BA−ph and WZ−cn polymers, cured under the aforementioned thermal profiles, was structured as follows: for instance, BA−ph subjected to a curing regimen of 180 °C−1 h/200 °C−2 h was denoted as BA−ph−200 °C. Similarly, BA−ph with the curing program of 180 °C−1 h/200 °C−2 h/220 °C−2 h was referred to as BA−ph−220 °C, and this pattern continued for the other samples.

### 3.3. Characterization

The proton nuclear magnetic resonance spectrum (^1^H-NMR) was obtained by the nuclear magnetic resonance spectrometer Bruker AV400 (NMR, Bruker, Karlsruhe, Germany) with deuterated DMSO as solvent at a proton frequency of 400 MHz.

Fourier transform infrared spectroscopy (FTIR) was recorded on a Vertex 70 spectrometer (Bruker, Billerica, MA, USA) for spectra between 500 and 4000 cm^−1^.

Differential scanning calorimetry analyses (DSC) were performed by modulating a DSC-Q100 (TA Instruments, New Castle, DE, USA) at a heating rate of 10 °C min^−1^ and a nitrogen flow rate of 50 mL min^−1^. DSC tests were performed on BA−ph and WZ−cn by heating the samples from 50 °C to 350 °C.

Thermogravimetric analysis (TGA) was performed on a ZRT-B (Beijing Jingyi Gaoke Instrument Co., Ltd., Beijing, China), with a heating rate of 20 °C min^−1^ (under nitrogen) and a purge rate of 40 mL min^−1^ from 50 °C to 800 °C.

Mechanical properties were measured by a SANS series bench-top electromechanical universal testing machine (CMT6104, Shenzhen, China) in three-point bending mode, with a test speed of 5 mm min^−1^ for crosshead displacement. The ratio of brace span to thickness was 15:1, and the results were obtained by averaging three samples. Refer to Chinese standard GB/T 2567-2008 [44].

The morphology of the fracture surface of the material was observed with a scanning electron microscope (SEM) phenom pharos G2 (Feiner, Amsterdam, The Netherlands) at a voltage of 20 kV.

Static thermodynamic analysis (TMA) was carried out using TMA Q400 (TA Instrument, New Castle, DE, USA) in an expansion mode from 50 °C to 350 °C in a static atmosphere with a heating rate of 10 °C min^−1^. The size of the samples was 5 mm × 5 mm × 1 mm. The samples were subjected to a force of 0.05 N in the TMA test. The linear coefficient of thermal expansion (CTE) of the sample was recorded.

### 3.4. Kinetic Analysis

Non-isothermal curing kinetics analysis of thermosetting resin systems is based on the following rate equations:

(3) $$\frac{d\alpha }{dt}=\beta \frac{d\alpha }{dT}=k(T)f(\alpha )$$

where α is the degree of curing reaction; f(α) is a function of α, determined by the curing reaction mechanism; β=dT/dt is a constant heating rate; k(T) is a temperature-dependent reaction rate constant and follows the Arrhenius equation.

(4) $$k\left(T\right)=Aexp\left(\frac{{-E}_{\alpha }}{RT}\right)$$

where *A* is pre-exponential factor; *E_α_* is apparent activation energy; *R* is gas constant; *T* is absolute temperature. By (1) and (2), non-isothermal kinetic parameters *E_α_* can be determined [20].

According to reports in the literature, the curing mechanism of thermosetting resins generally has two general kinetic reactions: an n-stage reaction model and an autocatalytic reaction model [21]. In the case of n-stage reactions, the kinetic equation is $\frac{d\alpha }{dt}=Aexp(\frac{-E_{\alpha }}{RT})f(\alpha )$, in which $f(\alpha )={\left(1-\alpha \right)}^{n}$.

In the case of an autocatalytic reaction model: $\frac{d\alpha }{dt}=Aexp\left(\frac{-E_{\alpha }}{RT}\right)f\left(\alpha \right)$, in which $f(\alpha )={\alpha^{m}\left(1-\alpha \right)}^{n}$.

## 4. Conclusions

In this work, a benzoxazine cyanide-based resin containing a fluorene structure (WZ−cn) was initially prepared. Through the analysis of polymerization reaction kinetics, the differences in reaction behavior and mechanism between it and the bisphenol A-type benzoxazine cyanide-based resin (BA−ph) were comparatively investigated. The results indicated that the introduction of the fluorene structure did not change the polymerization reaction mechanism of the resin, still demonstrating self-catalyzed polymerization. Nevertheless, the introduction of the fluorene structure enhanced the activation energy of the resin’s polymerization reaction. Subsequently, in situ infrared testing was utilized to monitor the transformation of the functional groups of WZ−cn and BA−ph monomers as the curing temperature rose, and the curing process of the resin system was determined in combination with the extrapolation method. Through the method of stepwise temperature rise curing, the cured samples of WZ−cn and BA−ph resins were respectively prepared. Through comparative analysis, it was discovered that there were significant differences in the heat resistance, structural strength, microscopic morphology, and dimensional stability of the benzoxazine cyanide-based resins with fluorene structure and bisphenol A structure at different thermal polymerization temperatures. The introduction of the fluorene structure increased the rigidity of the main chain structure and the spatial hindrance, leading to an increase in the difficulty of thermal polymerization and resulting in lower heat resistance of the polymer than the bisphenol A-structured cyanide-based resin. The bending strength and modulus of both polymers exhibited an increasing trend with the increase in the curing temperature. Among them, the bending strength and modulus of the WZ−cn polymer system were superior to those of BA−ph, which benefited from the higher molecular rigidity and matching curing conditions. Moreover, the influence law of heat treatment temperature conditions on the structure and properties of the polymer was analyzed. The microscopic morphology and dimensional stability of the two structural benzoxazine cyanide-based resin polymers were tested and analyzed, and it was found that both resin systems presented a rough and discontinuous granular microscopic morphology during the heat treatment process due to the segmented polymerization of functional groups. Additionally, the introduction of the fluorene structure significantly improved the dimensional stability of the benzoxazine cyanide-based resin.

## Figures and Tables

**Table 1:** DSC curve data for WZ−cn and BA−ph monomer.

Heating Rate (*β*, °C/min)	WZ−cn		BA−ph	
	Ttop1 (°C)	Ttop2 (°C)	Ttop1 (°C)	Ttop2 (°C)
5	232.9	265.0	216.0	248.4
10	243.4	277.8	228.7	263.3
15	252.7	288.4	236.5	272.6
20	258.8	294.1	243.9	280.3

**Table 2:** Reaction activation energies of WZ−cn and BA−ph.

Conversion Rate α	WZ−cn		BA−ph	
	Reaction 1 *E_α_* (KJ·mol^−1^)	Reaction 2 *E_α_* (KJ·mol^−1^)	Reaction 1 *E_α_* (KJ·mol^−1^)	Reaction 2 *E_α_* (KJ·mol^−1^)
0.1	116.0304764	94.4919994	87.72981315	84.58526902
0.2	117.5557066	95.98067726	89.63552018	86.44653803
0.3	118.6647384	97.03238829	91.02367906	87.77326917
0.4	119.6375296	97.92376739	92.24070643	88.90031012
0.5	120.5804143	98.756995	93.42367366	89.96682635
0.6	121.5548669	99.59686851	94.65482354	91.07508704
0.7	122.6306693	100.5040316	96.02802918	92.29718257
0.8	123.9166477	101.5740188	97.6895	93.74411739
0.9	125.7110362	103.099249	100.0396505	95.74797664
Average value	120.6980095	98.7733328	93.60726619	90.05961959

**Table 3:** The curing process temperature of WZ−cn.

*β* (°C/min)	*T_i_* (°C)	*T_p_* (°C)	*T_f_* (°C)
5	210.05	233.33	269.87
10	221.99	244.33	285.35
15	228.99	251.80	294.73
20	231.29	257.19	300.89
0 (Extrapolated temperature)	205.40	226.90	262.10

**Table 4:** The curing process temperature of BA−ph.

*β* (°C/min)	*T_i_* (°C)	*T_p_* (°C)	*T_f_* (°C)
5	189.58	216.27	256.82
10	203.22	228.32	273.32
15	213.46	236.45	282.48
20	221.28	244.16	290.16
0 (Extrapolated temperature)	180.55	208.35	248.40

**Table 5:** TGA data of BA−ph and Wz−cn polymers.

Samples	*T*_5%_ (°C)	*T*_10%_ (°C)	*Y_C_* (%, 600 °C)
BA−ph−200	380.74	428.49	74.37
BA−ph−220	382.16	437.9	76.51
BA−ph−240	399.12	451.71	78.59
WZ−cn−200	237.39	285.39	66.88
WZ−cn−220	307.42	388.44	74.72
WZ−cn−240	321.09	396.49	74.44

**Table 6:** Thermal expansion performance parameters of various polymers.

Samples	Expansion Rate (%, 250 °C)	CTE (ppm/°C, 50−250 °C)
WZ−cn−200	−0.42105	−18.81
WZ−cn−220	1.43147	67.92
WZ−cn−240	0.58628	29.31
BA−ph−200	28.53238	1091
BA−ph−220	6.74433	334
BA−ph−240	1.17285	54.24

## Data Availability

The raw/processed data required to reproduce these findings cannot be shared at this time as the data also forms part of an ongoing study.
